# Validation of the Comprehensive and Brief International Classification of Functioning, Disability and Health Core Sets for Schizophrenia from the Perspective of Individuals Diagnosed with the Disorder: A Worldwide Study Using Focus Groups

**DOI:** 10.3390/bs14111032

**Published:** 2024-11-04

**Authors:** Chuen Ann Chai, Maite Barrios, Juana Gómez-Benito, Karina Campoverde, Georgina Guilera

**Affiliations:** 1 Department of Social Psychology and Quantitative Psychology, Faculty of Psychology, University of Barcelona, 08035 Barcelona, Spain; chuenchai@ub.edu (C.A.C.); juanagomez@ub.edu (J.G.-B.); karina2529@gmail.com (K.C.); gguilera@ub.edu (G.G.); 2 Group on Measurement Invariance and Analysis of Change (GEIMAC), Institute of Neuroscience, University of Barcelona, 08035 Barcelona, Spain; 3 Centre Psicoterapia Barcelona-Serveis Salut Mental (CPB-SSM), 08008 Barcelona, Spain

**Keywords:** schizophrenia, functioning, validation, focus groups, ICF core set

## Abstract

The comprehensive and brief International Classification of Functioning, Disability and Health (ICF) core sets for schizophrenia, based on the World Health Organization (WHO) framework, aim to describe the functioning of individuals with schizophrenia. The objective of this study was to identify the most common problems faced by these individuals and validate the ICF core sets. Eight focus groups were conducted, recorded, and transcribed verbatim. The linking process involved two independent coders identifying meaningful units and linking agreed-upon concepts to the ICF categories. Data saturation was defined as the point at which no new categories emerged from additional focus groups. The 37 participants in this study represented the WHO regions of Africa, South-East Asia, the Western Pacific, and Europe. The focus groups confirmed the relevance of all ICF core set categories, with an additional 21 second-level categories being proposed in at least six of the eight focus groups. In this study, the ICF core sets for schizophrenia were validated from the perspective of individuals. However, several second-level categories not currently included in the ICF core sets also emerged. To ensure that the ICF core sets are truly international in scope, the potential relevance of these categories should be investigated further.

## 1. Introduction

Schizophrenia is a prevalent mental disorder that impacts approximately 1% of the global population [1,2,3] and manifests through a spectrum of symptoms, such as hallucinations, delusions, negative symptoms, anhedonia, social withdrawal, and cognitive impairment [4,5]. This symptomatology can significantly impair daily functioning, hindering an individual’s ability to be independent and live a fulfilling life [6].

Recognizing the need to enhance the understanding of functioning in any health condition, in 2001, the World Health Organization (WHO) developed the International Classification of Functioning, Disability and Health [7]. This classification system is based on a biopsychosocial model of disability and provides a comprehensive framework for categorizing health-related domains. The ICF is structured into the following components: *Body Functions*, *Body Structures*, and *Activities and Participation*, along with two contextual components, *Environmental factors* and *Personal factors*. It encompasses over 1400 categories organized in a hierarchical classification system, with each category being identified by an alphanumeric code: b for *Body Functions*, s for *Body Structures*, d for *Activities and Participation*, and e for *Environmental Factors*, followed by a numeric code. Each numeric code is structured so as to reflect increasing levels of detail, beginning with broad chapters (groupings) and moving on to more specific categories. The chapter number (one digit) represents the broadest level of classification. This is followed by the second level (two digits), which provides more detail within each chapter, and subsequently by the third (one digit) and fourth (one digit) levels. The higher the level, the more specific the categories become, providing finer detail with respect to the aspect being evaluated.

Because the extensive number of categories in the ICF makes it cumbersome and impractical in clinical settings, a series of ICF core sets (ICF-CSs) were developed with the aim of streamlining its application and facilitating a systematic and comprehensive description of functioning and disability in practice. There are two types of ICF-CSs, comprehensive and brief. Comprehensive ICF-CSs encompass the ICF categories that enable a comprehensive and exhaustive description of functioning for a given health condition, while brief ICF-CSs include only the most essential categories, the fewest possible number required to serve as the minimal standard for describing functioning [8]. The ICF-CSs for schizophrenia [9] were established according to the standard multi-method process for developing an ICF-CS [8]. Specifically, the process involved the following four preparatory studies: (1) a systematic literature review analyzing 206 studies on the functioning of people with schizophrenia, which identified and quantified the main concepts [10]; (2) a qualitative study with focus groups comprising individuals with schizophrenia and their relatives, providing a comprehensive perspective of functionality [11]; (3) an expert survey involving 189 participants from all WHO regions and various disciplines, which identified ICF categories, predominantly in *Body Functions*, *Activities and Participation*, and *Environmental Factors* [12]; and (4) a multicenter study evaluating the clinical perspective by identifying the most frequently mentioned functioning problems [13]. The results of these studies were the starting point for a structured decision-making and consensus process at an international conference, a process that included 20 health professionals from around the world with expertise in the field of schizophrenia, who determined the categories to be included in the comprehensive and brief ICF-CSs for schizophrenia [9]. The resulting comprehensive ICF-CS for schizophrenia comprises 97 second-level categories, as follows: 17 from *Body Functions*, 48 from *Activities and Participation*, and 32 from *Environmental Factors*. The corresponding brief ICF-CS includes a condensed selection of 25 of these categories, as follows: 8 from *Body Functions*, 10 from *Activities and Participation*, and 7 from *Environmental Factors*. This selection still captures the essential aspects of functioning and disability of relevance to this condition.

The comprehensive and brief ICF-CSs for schizophrenia have undergone rigorous global testing and validation using the Delphi method with health professionals from across the WHO regions (i.e., Africa, the Americas, South-East Asia, Europe, Eastern Mediterranean, and the Western Pacific). Contributions from psychiatrists [14], psychologists [15], psychiatric-mental-health nurses [16], occupational therapists [17], social workers [18], and physiotherapists [19] have been integral to the validation process. While the validation of the comprehensive and brief ICF-CSs for schizophrenia has received widespread acknowledgement from health professionals, the perspective of individuals with schizophrenia themselves has yet to form part of this validation process. Their input is crucial, however, in ensuring that the ICF-CSs for schizophrenia accurately reflect the functioning and challenges faced by people with this health condition.

To address this gap, in the present study, qualitative research methods were employed to capture the patient perspective in depth. Specifically, focus groups were used to gain detailed insights into patients’ experiences, providing rich data that enhance understanding of their health experiences. The aim of this study was twofold, first, to identify the common functioning problems faced by individuals with schizophrenia across diverse WHO regions, and second, to validate the ICF-CSs for schizophrenia from the perspective of individuals with this health condition.

## 2. Methods

### 2.1. Study Design

A cross-sectional, multicenter qualitative study employing focus group methodology was used to explore the perspectives of individuals living with schizophrenia. This study considered the complete range of ICF categories so as to accurately represent the perspectives of these individuals with regard to their functioning, and it aimed to validate both the comprehensive and brief ICF-CSs for schizophrenia.

This study was approved by the Bioethics Commission of the University of Barcelona (IRB00003099) and received ethical approval from all participating centers, while also conforming to the standards of the Helsinki Declaration.

### 2.2. Participants

Eligible participants were recruited from different mental health centers and hospitals in Equatorial Guinea (for Africa), India (for South-East Asia), Malaysia (for the Western Pacific), and Spain (for Europe). Inclusion criteria were age 18 or over and a primary diagnosis of schizophrenia according to either the Diagnostic and Statistical Manual of Mental Disorders (DSM-V) [20] or the International Classification of Diseases (ICD-11) [21]. Individuals with a primary diagnosis of a mental disorder other than schizophrenia, who had a serious medical or neurological pathology, a surgical wound that had not fully healed, or who presented intellectual disability, sensory impairment, acute positive symptoms, or severe cognitive impairment were excluded.

Purposeful sampling was used to ensure that participants could adequately contribute to the focus group. Mental health professionals from each center or hospital approached individuals who met the inclusion criteria and invited them to participate in the focus group study. Those who agreed to take part signed an informed consent form and stated their availability for a focus group session. Focus group sessions were then scheduled for a specific day and time at which a minimum of four participants had confirmed their availability, with participants being assigned to their respective groups.

### 2.3. Data Collection

After obtaining informed consent, we gathered sociodemographic and clinical data from participants, namely age, gender, current living arrangement, marital status, primary occupation, date of diagnosis, type of patient care (i.e., outpatient, day clinic, inpatient), and major medication use.

Focus groups were conducted either face-to-face or online. Online and face-to-face focus groups yield data of comparable quality, and hence both modalities were employed to facilitate and encourage participation [22]. Each focus group comprised four to seven participants, which is considered the ideal group size for facilitating discussion and interaction among participants [23,24].

The face-to-face focus groups were conducted at mental health centers or hospitals in each respective country, while the online groups were held via Zoom. Both focus group modalities were led and facilitated by at least one moderator, while a field assistant took notes and observed interactions. The moderators had expertise in the field of schizophrenia and adhered to the standardized procedures for conducting focus groups outlined [25]. At the beginning of each focus group, the moderator provided an introduction, explained the procedure and technical aspects in lay terms, and presented the six open-ended questions addressing ICF components and the questions related to each ICF chapter which is presented in Supplementary Table S1. Participants were asked all the questions in the topic guide verbally by the moderator, and each question was accompanied by visual support. Participants were encouraged to discuss their own views of coping with schizophrenia, and when needed, the moderator probed further using follow-up questions to elicit additional explanations. The online focus groups were video recorded through Zoom, while the face-to-face focus groups were digitally recorded. The recordings were then transcribed verbatim.

### 2.4. Data Analysis

#### Qualitative Analysis and Linking to the ICF

The qualitative analysis was carried out by two independent health professionals with experience in applying the ICF, using the meaning condensation procedure [26]. In a first step, the two coders independently read the focus group transcripts to gain an overall view and perspective. The second step entailed content analysis to determine meaningful units, which represented common themes present in participants’ utterances, regardless of linguistic or grammatical rules. Lastly, the two coders independently linked all responses to the corresponding second-level ICF categories using established linking rules [27,28,29]. Each individual concept could be associated with one or more ICF categories. For instance, when a moderator inquired about the *Body Functions* component with the question ’*Thinking of your body and mind, what does not work the way it is supposed to?*’, one participant responded ’*I am not thinking very fast*’. Here, the extracted concept was ’thinking,’ which was then assigned to *b160 Thought functions*. Any discrepancies between the two coders were reviewed and discussed by the research team to reach a consensus.

The degree of agreement between the two coders regarding the linked ICF categories was determined by calculating the Kappa statistic with a 95% confidence interval [30]. Values of the Kappa coefficient range from 0 to 1, with 0 indicating no agreement and 1 indicating perfect agreement.

### 2.5. Data Saturation

Data saturation occurs when additional data collection fails to provide new information and predetermined codes or themes are well represented in the data [31]. In the present study, saturation was defined as the point at which no new second-level ICF categories emerged from additional focus groups. This was determined when each category had been addressed by at least one focus group.

#### Correspondence Between Focus Group Responses and the ICF-CS for Schizophrenia

The categories identified in the focus groups were compared with those featured in the comprehensive and brief ICF-CSs for schizophrenia. This comparison helped establish content-related validity for the ICF-CSs by confirming the correspondence between the focus group data and the categories represented in the ICF-CSs. In addition, newly identified second-level categories (i.e., not currently included in the comprehensive ICF-CS for schizophrenia) were recorded when they appeared in at least six focus groups, representing 75% of all groups conducted.

## 3. Results

### 3.1. Description of the Focus Groups

A total of thirty-seven participants took part in the eight focus groups that were conducted across the four WHO regions: one focus group in Equatorial Guinea (*n* = 4 participants), two in India (*n* = 12), three in Malaysia (*n* = 12 participants), and two in Spain (*n* = 9 participants). Participants’ demographic and clinical characteristics are summarized in Table 1.

### 3.2. Qualitative Analysis and Linking

A total of 1300 concepts were identified in the focus groups, and these concepts were linked to 193 unique second-level ICF categories. Ninety concepts could not be linked to specific ICF categories, examples being those referring to other health conditions (e.g., depression), or not covered by the ICF system (e.g., suicide), or which were too generic to be linked to a specific category (e.g., medication side effects). Other concepts that could not be linked related to personal factors (e.g., age, personality) which are not classified by the ICF. The Kappa coefficient for agreement between the two coders was 0.89 with a 95% CI [0.74, 1.03].

Data saturation was reached after the seventh focus group, as the eighth group did not yield any additional second-level ICF categories.

### 3.3. Confirmation of the ICF Core Sets for Schizophrenia

All 97 categories included in the comprehensive ICF-CS for schizophrenia emerged in the focus groups. Specifically, 48 categories (49.5%) were validated by all eight groups, while another 23 categories (23.7%) were confirmed by seven groups. Of the remaining categories, fourteen categories (14.4%) were confirmed by six groups, six (6.2%) by five groups, two (2.1%) by four groups, two (2.1%) by two groups, and two (2.1%) by one group.

All 25 categories in the brief ICF-CS for schizophrenia were confirmed. Of these, 16 categories (64.0%) were identified across all eight focus groups. Of the remaining categories, five (20.0%) were identified by seven groups, three (12.0%) by six groups, and one (4.0%) by one group. The complete list of categories from the comprehensive and brief ICF-CSs for schizophrenia that were reported by each focus group can be consulted in the Supplementary Table S2.

### 3.4. Additional Categories Not Included in the Comprehensive ICF-CS for Schizophrenia

A total of 21 second-level ICF categories not currently included in the comprehensive ICF-CS for schizophrenia were identified in six or more focus groups, and hence they emerged in at least 75% of the groups conducted. These additional categories predominantly pertained to the *Environmental Factors* component, with 10 categories (47.6%), followed by *Body Functions* and *Activities and Participation* with 5 categories (23.8%) each. One of the additional categories (4.7%) related to *Body Structures*. Table 2 presents the additional ICF categories identified in six or more focus groups (i.e., representing 75% of all groups conducted), specifying the country where they were reported and the total number of focus groups in which each ICF category arose. The full list of the additional categories that emerged in the focus groups is available in the Supplementary Table S3.

## 4. Discussion

### 4.1. Validation of the ICF-CSs for Schizophrenia

In this study, the functioning problems of individuals diagnosed with schizophrenia were identified from their own perspective, the aim being to provide content-based validity evidence for the comprehensive and brief ICF-CSs for schizophrenia. The results obtained through the active participation of individuals with schizophrenia from several WHO regions (Africa, South-East Asia, the Western Pacific, and Europe) provide compelling support for both these core sets. Specifically, all 97 categories of the comprehensive ICF-CS for schizophrenia—and by extension, the 25 categories from the brief version—were confirmed.

To date, the ICF-CSs for schizophrenia have been validated exclusively from the perspective of health professionals [32]. In this respect, the present research expands upon existing validation studies of the ICF-CSs by incorporating for the first time the perspective of individuals diagnosed with schizophrenia.

Categories relating to the *Body Functions* component of the ICF-CSs emerged in all eight focus groups. Those categories referring to negative symptoms (i.e., *b130 Energy and drive functions*, *b152 Emotional functions*) warrant particular attention as they reflect the other findings of Correll and Schooler [5], who highlighted the persistence of these symptoms throughout the course of schizophrenia. Their review reports that 60% of individuals with schizophrenia exhibit prominent or predominant negative symptoms, which frequently remain clinically significant despite treatment efforts. It is important to note that although antipsychotic medications, particularly long acting injectables, have been shown to improve the quality of life for individuals with schizophrenia, their effects are often underrepresented in functional assessments [33]. In light of this, Galderisi et al. [34] stress the imperative to prioritize both pharmacological and psychosocial interventions, as addressing negative symptoms remains a critical and unmet challenge in the management of schizophrenia.

Similarly, categories associated with positive symptoms (i.e., *b160 Thought functions*, *b156 Perceptual functions*) within the *Body Functions* component of the ICF-CSs were consistently identified across all focus groups. Notably, this occurred despite all participants being on a treatment regimen that included at least one antipsychotic drug to mitigate these positive symptoms. Although the efficacy of antipsychotics in reducing the positive symptoms of schizophrenia has been well-documented [35,36], and despite evidence that their administration can lead to symptom and functional remission [37], our finding here suggests that the impact of positive symptoms on individuals may persist, even in the context of pharmacological intervention and during a remission phase.

Categories related to cognitive deficits (i.e., *b164 Higher-level cognitive functions*, *b180 Experience of self and time functions*) within the *Body Functions* component of the ICF-CSs were also identified across all focus groups. McCutcheon, Keefe, and McGuire [38] note that this core feature of schizophrenia contributes significantly to the impaired functioning observed in affected individuals, with current pharmacological treatments being largely ineffective in addressing these cognitive challenges. This aligns with the subjective reports from participants in the focus groups, who identified cognitive deficits as a major concern. These findings underscore the potential value of behavioral training-based interventions, particularly cognitive remediation therapy, in mitigating cognitive difficulties and promoting recovery [39,40]. However, access to such therapies remains limited, especially in low-income settings where availability lags behind that of middle- and high-income regions [41].

Regarding Activities and Participation, all eight focus groups identified several categories from the ICF-CSs, particularly d230 Carrying out daily routine, d570 Looking after one’s health, d720 Complex interpersonal interactions, and d760 Family relationships. This concurs with the perspective of health professionals from diverse backgrounds who play a significant role in the treatment of individuals with schizophrenia [32]. Previous studies have shown that category d230 Carrying out daily routine reflects a significant challenge for individuals with schizophrenia. For example, Høier et al. [42] found that these individuals often experience a lack of structure and engage in very few activities during the day. Similarly, Schneider et al. [43] reported that individuals with schizophrenia often spend time doing nothing. These findings, together with the results from our focus groups, highlight the need to support individuals with schizophrenia in engaging in meaningful activities that hold personal value and generate positive emotions [44]. Providing such support could potentially improve their daily routine.

The category *d570 Looking after one’s health* emerged in all the focus groups, underlining the extent to which this is also a challenge for individuals with schizophrenia. In particular, participants reported finding it difficult to maintain a healthy diet and remain active, reflecting the findings of Kalinowska et al. [45], who noted that individuals with schizophrenia often have poor diets and engage in insufficient physical activity. The implications of this are evidenced by the fact that participants in all eight focus groups complained about being overweight or obese, thus validating the category *b530 Weight maintenance functions* that features in the comprehensive ICF-CS for schizophrenia. The category *d720 Complex interpersonal interactions* was also identified in all eight focus groups. Participants frequently reported difficulties related to this category, particularly as regards forming and maintaining relationships. This is reflected in the fact that the majority of them were single and spoke of struggling to find a romantic partner. This aligns with the findings of Budziszewska, Babiuch-Hall, and Wielebska [46], who noted that individuals with schizophrenia encounter internal challenges due to illness-induced changes. These changes significantly impact their experiences of love and serve as obstacles to forming and maintaining relationships. Additionally, these individuals often struggle to comprehend others’ intentions, further complicating interpersonal interactions.

Individuals with schizophrenia tend to rely heavily on family members during the course of their illness [47]. The category *d760 Family relationships*, which is included in both the comprehensive and brief ICF-CSs for schizophrenia, emerged in all eight focus groups, with participants reporting frequent interactions with their family members. Consistent with this, the study by Weittenhiller et al. [48] found that individuals with schizophrenia were less likely than those without the condition to spend time with friends, but not with family members. However, the behavior caused by the disorder often creates tension in family relationships. Fernandes et al. [49] suggest that this tension is the result of several factors, including the behavior of individuals with schizophrenia and the cultural and social stigma associated with the disease, which complicates adaptation to the caregiver role.

Overall, our findings in relation to the *Activities and Participation* component of the ICF-CSs for schizophrenia underscore the substantial disruption of daily functioning that the disorder causes in these areas. Consequently, treatment strategies should prioritize the enhancement of psychosocial functioning so as to enable individuals to actively engage and participate in these essential aspects of life [50].

Consistent with the perspective of health professionals [32], the views of focus group participants underline the critical role of *Environmental Factors*, including both facilitators and barriers, in the context of schizophrenia. In the comprehensive and brief ICF-CSs for schizophrenia, this recognition encompasses various aspects, such as the significance of support and relationships (e.g., *e310 Immediate family*, *e355 Health professionals*) and the attitudes of individuals in the social environment (e.g., *e410 Individual attitudes of immediate family members*, *e450 Individual attitudes of health professionals*, *e460 Societal attitudes*). Participants here often emphasized the support they received from family members as caregivers and facilitators in their recovery. Clearly et al. [51] and Kim and Park [52] stressed that family caregivers play an important role in the improvement of individuals with schizophrenia, contributing to their quality of life and well-being. However, Kim and Park [52] also showed that family caregivers can be a barrier, insofar as strongly expressed emotions—such as critical comments, emotional over-involvement, and hostility—may increase relapse rates among individuals with schizophrenia. This relational dynamic was evident in our findings, as family interactions of this kind were also identified as a barrier.

The participants also emphasized the role of societal attitudes as either facilitators or barriers with respect to their disorder. For example, participants in some of the focus groups reported experiencing public stigma, including negative stereotypes such as being perceived as violent. The presence of this negative stereotype is supported by a systematic review and meta-analysis by Degnan et al. [53], which found that individuals diagnosed with schizophrenia are often viewed by the public as highly dangerous and aggressive. However, our participants also acknowledged that there is now greater social awareness of mental disorders, partly due to anti-stigma campaigns, which have served as a facilitator for these individuals.

Another issue that emerged in our focus groups concerned the role of social security services (*e570 Social security services, systems and policies*) in enabling individuals with schizophrenia to access benefits and resources so as to improve their functioning, a view fully supported by health professionals [32]. Some participants reported receiving significant government aid. In this regard, Simpson et al. [54] found that expanding social security improves mental health by providing additional income, although their study focused on high-income countries, suggesting the need for similar benefits in all income settings.

Another category that emerged in all eight focus groups, and which fully aligns with the perspective of health professionals [32], concerned the accessibility of health services (e.g., *e580 Health services, systems and policies*). Participants reported having access to necessary health services, including hospitals and mental health facilities, which facilitated their treatment and care. However, limited access to health services was a notable barrier in Equatorial Guinea, where individuals often had to travel long distances to receive appropriate treatment, highlighting a critical gap in the country’s healthcare infrastructure.

### 4.2. Additional ICF Categories Identified from the Focus Groups

A total of 21 additional ICF categories (i.e., not currently included in the comprehensive ICF-CS for schizophrenia) were identified across six to eight focus groups. Regarding *Body Functions*, the categories *b167 Mental functions of language* and *b760 Control of voluntary movement functions* emerged in all focus groups. The relevance of the language functions aligns with findings from other studies indicating common language deficits in verbal ability and fluency among individuals with schizophrenia [2,55]. Similarly, deficits in the control of voluntary movements are well-documented, with movement disorders such as akathisia, dyskinesia, dystonia, and parkinsonism—often linked to low physical activity and sedentary behavior—being highly prevalent in this population and significantly impairing their functioning and clinical outcomes [56].

An additional category, not included in the comprehensive ICF-CS for schizophrenia and supported by seven groups, is *b126 Temperament and personality*. In this context, a systematic review by Franquillo et al. [57] highlighted the importance of personality traits in individuals with schizophrenia. Their study found that low levels of extraversion were linked to lower perceived quality of life, while high neuroticism and low extraversion were associated with a longer duration of untreated psychosis and an increased risk of developing schizophrenia. These findings suggest that personality traits can profoundly impact mental health outcomes and overall functioning in individuals with schizophrenia. Notably, studies of health professionals’ perspectives [32] also emphasized the importance of this category, underscoring the relevance of personality in overall functioning.

The identification of category *b280 Sensation of pain* as an additional category was linked to participants reporting pain in areas such as the head, stomach, chest, and knees. However, this finding is not supported by the perspectives of health professionals [32]. The prominence of this category may be attributable to other health-related conditions affecting our participants. It is worth noting here that it is difficult to objectively measure pain, and studies present contradictory findings—some suggest an increased pain threshold in individuals with schizophrenia, others indicate heightened pain sensitivity, and some have found no clear relationship between pain and schizophrenia [58].

The category *b455 Exercise tolerance* also emerged as a significant concern among participants. In this regard, a systematic review and meta-analysis by Firth et al. [59] documented how individuals with schizophrenia face both physical health barriers, such as tiredness, and psychological barriers, including lack of motivation, to a greater extent than do healthy controls. However, it is crucial for individuals with schizophrenia to engage in exercise. As noted by several studies [60,61], exercise can be considered an important adjunct therapy in managing schizophrenia, particularly for improving physical health and reducing negative symptoms, which are often difficult to treat with medication alone. Incorporating exercise or strategies to increase exercise tolerance should therefore be considered a beneficial component of treatment plans for schizophrenia.

Although the comprehensive ICF-CS for schizophrenia does not currently include categories relating to *Body Structures*, the category *s530 Structure of stomach* did emerge in the majority of focus groups. Participants reported experiencing pain in this area, as well as gastroesophageal reflux. This aligns with the findings of Colijn [62], who noted that individuals with schizophrenia commonly experience gastrointestinal issues for a variety of reasons, including foreign body ingestion, somatoform, or delusional influences, or medication side effects. These observations highlight the importance of considering gastrointestinal structures when assessing the functioning of individuals with schizophrenia.

In *Activities and Participation*, seven focus groups reported on several categories that merit attention (*d170 Writing, d550 Eating, d810 Informal education, d940 Human rights*) but which are not currently part of the ICF-CS for schizophrenia. The additional category *d170 Writing* may stem from deficits in language comprehension, as indicated by Vanova et al. [63]. Concerning *d550 Eating*, individuals reported notable functional challenges. Several studies [64,65,66] consistently demonstrated that individuals with schizophrenia often exhibit unhealthy dietary patterns, characterized by high saturated fat and calorie intake alongside inadequate fiber and fruit consumption. The relevance of the category *d810 Informal education* is reflected in the employment-related challenges encountered by many individuals in the focus groups. This observation aligns with the study by Harvey, Strassnig, and Silberstein [67], which found that even with adequate antipsychotic treatment, individuals with schizophrenia continue to struggle with maintaining employment.

The emergence in the majority of focus groups of category *d940 Human rights* highlights the vulnerability of individuals with schizophrenia to human rights violations, such as involuntary confinement. This concerning trend is reflected in the findings of a recent systematic review by Wigand et al. [68] and a scoping review by Schomerus et al. [69], both of which noted the prevalence of human rights abuses in this population. Our participants also reported instances of abuse, including physical and verbal harassment, which is likewise consistent with the findings of Wang et al. [70] in China and the results of a narrative review by González-Rodríguez et al. [71].

As a result of technological advancements, the category *d360 Using communication devices and techniques* has become increasingly relevant to contemporary life, and this is evidenced by the emergence of this additional category in six of the eight focus groups. The cognitive difficulties experienced by many individuals with schizophrenia can make it difficult for them to use communication devices and techniques effectively. Negative symptoms may also impede their ability to engage with these devices or benefit from them. Research by Sunil, Sharma, and Amudhan [72] and Athanasopoulou et al. [73] has highlighted the difficulties faced by individuals with schizophrenia spectrum disorder when it comes to using the internet, primarily due to attentional and motivational deficits. Sunil, Sharma, and Amudhan [72] further elaborate on how negative symptoms and paranoid ideation associated with the disorder contribute to reduced internet use.

The largest number of additional categories related to *Environmental factors*. Three pertained to the chapter e1 Products and Technology (i.e., *e115 Products and technology for personal use in daily living*, *e135 Products and technology for employment*, e140 Products and technology for culture, recreation, and sport), highlighting how individuals with schizophrenia regard the use of products and technology as highly relevant to their daily lives, as these categories were discussed as potentially being both barriers and facilitators. Consistent with this view, a study in Taiwan [74] found that more than 30% of individuals with schizophrenia identified the categories of chapter e1 Products and Technology as a barrier. This underscores the importance of providing a barrier-free environment to reduce disability among individuals with schizophrenia.

The other seven additional categories relating to *Environmental factors* (i.e., *e515 Architecture and construction* services, systems and policies, *e520 Open space planning services, systems and policies*, *e530 Utilities services, systems and policies*, *e535 Communication services, systems and policies*, *e540 Transportation services, systems and policies*, *e565 Economic services, systems and policies*, and *e595 Political services, systems and policies*) all pertain to chapter *e5 Services, systems, and policies*. This suggests that individuals with schizophrenia recognize how the provision of services impacts their overall functioning. Vera San Juan et al. [75] similarly showed that the optimal provision of services can aid in both clinical and personal recovery.

The present research has several strengths. To our knowledge, this is the first validation study of an ICF-CS to be conducted across multiple countries from diverse WHO regions (Africa, South-East Asia, the Western Pacific, and Europe) using focus groups [76]. Furthermore, it is the first to validate the ICF-CSs for schizophrenia from the perspective of individuals with this health condition. In addition to confirming existing ICF categories, this study identified additional categories of relevance to individuals with schizophrenia, thus contributing to a more comprehensive understanding of their functioning and the challenges they face. The generalizability of the findings to both different cultural settings and the broader population of individuals with schizophrenia is supported by the inclusion of participants from Equatorial Guinea, India, Malaysia, and Spain, as well as the fact that the sample comprised both recently diagnosed individuals and those with a longstanding diagnosis. Furthermore, the use of focus groups to gather data directly from individuals with schizophrenia ensures that the findings are grounded in the real-world experiences of the target population.

The methodological rigor of this study is reflected in the meticulous use of multiple coders during the linking process. Discrepancies among coders were resolved by consensus in consultation with the research team, resulting in a Kappa coefficient that indicated almost perfect agreement. The focus groups comprised between four and seven participants, striking a balance that fostered engagement and facilitated meaningful data collection on this sensitive topic [23]. This number of participants is optimal because larger groups may inhibit their willingness to speak. Data saturation was achieved as the eighth focus group did not produce any additional second-level ICF categories, demonstrating that further focus groups would be redundant.

One limitation of this study is the absence of representation from the Americas and Eastern Mediterranean WHO regions, despite including individuals with schizophrenia from other regions. Future research should aim to include participants from these regions to provide a more comprehensive global perspective on the validation of the ICF-CSs for schizophrenia. Additionally, the use of purposive sampling, with participants recruited by mental health professionals from specific centers, introduces the possibility of selection bias. Given that mental health professionals selected participants based on their own judgement and familiarity with individuals who met the inclusion criteria, it is possible that they may have favored certain individuals over others, consciously or unconsciously. This process may have resulted in the inclusion of participants with particular characteristics, such as those who were more engaged in treatment, more articulate, or more comfortable with the professionals, potentially skewing the data toward a certain perspective. This selection bias could limit the diversity of experiences and opinions represented in the focus groups, affecting the range of insights gathered. As a result, the findings may not fully reflect the broader population of individuals who meet the inclusion criteria, particularly those who might be less connected to mental health services or less willing to engage in group discussions. Consequently, this limitation should be considered when interpreting the generalizability of this study’s results. Future research should also consider integrating the perspectives of caregivers through focus groups across all WHO regions, as their insights could provide valuable information on the functioning and support needs of individuals with schizophrenia, thereby contributing to a more comprehensive understanding of the disorder.

## 5. Conclusions

In this study, a cross-cultural perspective is offered on individuals with schizophrenia and validates both the comprehensive and brief ICF-CSs for this health condition, confirming their applicability across diverse WHO regions. The emergence of additional categories in the focus groups underscores the need to consider these aspects when assessing functioning in individuals with schizophrenia. Further research should incorporate caregiver perspectives though as their insights could further illuminate the additional ICF categories identified, contributing to a more comprehensive understanding of the functioning and support needs of individuals with schizophrenia and enhance clinical practice.

## Figures and Tables

**Table 1:** Demographic and clinical characteristics of participants.

	Equatorial Guinea	India	Malaysia	Spain	All Countries
Age *M (SD)*, *[Range]*	37.5 (7.19), [27–43]	43.92 (13.70), [24–63]	41.08 (13.57), [22–64]	47.67 (6.36), [41–60]	43.22 (11.71), [22–64]
Gender (male/female)	1/3	8/4	9/3	5/4	23/14
Current living arrangement					
Family of origin	1	3	9	3	16
Own family			1	1	2
Partner/Spouse	3	2			5
Alone			1	1	2
Residential care		7	1		8
Roommates				4	4
Marital status					
Single	1	5	11	4 ^a^	21
Married	3	3			6
Separated/ Divorced		3		2	5
Widowed		1	1		2
Primary occupation					
Student		1			1
Volunteer work		1		1	2
Paid employment	1		2		3
Homemaker	3				3
Retired			1	1	2
Unemployed		10	9	7	26
Type of patient care					
Outpatient (ambulatory)	4	3	2	9	18
Day clinic patient		4	5		9
Inpatient		5	5		10

Note. ^a^ In Spain, data on ’marital status’ are missing for three participants due to psychotic episodes. All participants were being treated with at least one antipsychotic drug. In addition to antipsychotics, some participants were prescribed benzodiazepines to manage anxiety and agitation, as well as antidepressants to address co-occurring depressive symptoms.

**Table 2:** Additional ICF categories reported across six or more focus groups.

ICF Category	Equatorial Guinea	India	Malaysia	Spain	Total Groups
Focus groups	*n* = 1	*n* = 2	*n* = 3	*n* = 2	
*Body functions*					
b126 Temperament and personality functions	1	1	3	2	7
b167 Mental functions of language	1	2	3	2	8
b280 Sensation of pain	1	2	1	1	6
b455 Exercise tolerance functions	1	1	2	1	6
b760 Control of voluntary movement functions	1	2	3	2	8
*Body structures*					
s530 Structure of stomach	0	2	2	2	6
*Activities and participation*					
d170 Writing	1	2	2	2	7
d360 Using communication devices and techniques	1	2	2	1	6
d550 Eating	1	2	3	1	7
d810 Informal education	0	2	3	2	7
d940 Human rights	1	1	3	2	7
*Environmental factors*					
e115 Products and technology for personal use in daily living	1	2	3	2	8
e135 Products and technology for employment	1	2	3	2	8
e140 Products and technology for culture, recreation and sport	1	2	3	2	8
e515 Architecture and construction services, systems and policies	1	1	3	1	6
e520 Open space planning services, systems and policies	1	2	3	2	8
e530 Utilities services, systems and policies	1	2	3	2	8
e535 Communication services, systems and policies	1	2	3	2	8
e540 Transportation services, systems and policies	1	2	3	2	8
e565 Economic services, systems and policies	1	2	3	2	8
e595 Political services, systems and policies	1	2	3	0	6

## Data Availability

The data that support findings of this study are available from the corresponding author upon reasonable request.

## Supplementary Materials

The following supporting information can be downloaded at: https://www.mdpi.com/article/10.3390/bs14111032/s1, Table S1: The six open-ended questions addressing ICF components, and the questions related to each ICF chapter. Table S2: Categories included in the Comprehensive ICF-CS for schizophrenia that were reported across focus groups. Table S3: Additional ICF categories reported across focus groups.
