# Supplementary material for: Validation of the Comprehensive and Brief International Classification of Functioning, Disability and Health Core Sets for Schizophrenia from the Perspective of Individuals Diagnosed with the Disorder: A Worldwide Study Using Focus Groups
Source: Behav Sci (Basel). 2024 Nov 4;14(11):1032. doi: 10.3390/bs14111032 (PMC11591151; doi:10.3390/bs14111032)
Supplement: Supplementary file 1 [file behavsci-14-01032-s001.zip › behavsci-3252541-supplementary.pdf]

Supplementary Material

Table S1.

The six open-ended questions addressing ICF components, and the questions related to each ICF chapter.

ICF Components	The six open-ended ICF component questions	The questions related to each ICF chapter
Body Functions	Thinking of your body and mind, what does not work the way it is supposed to?	Mental functions: This area involves everything having to do with mental functions such as orientation, consciousness, memory, attention, thought. What are your problems in this area?<br>Voice and Speech functions: This area involves all aspects having to do with the way you talk, such as the production of sounds and speech. What are your problems in this area?<br>Functions of the digestive, metabolic and endocrine systems: This area involves all aspects of your digestion and weight. What are your problems in this area?<br>Genitourinary and reproductive functions: This area involves all aspects of urination functions, reproductive functions and sexual relationships. What are your problems in this area?<br>Neuromusculoskeletal and movement-related functions: This area involves all aspects of the way you move and the mobility of joints, bones, muscles and reflexes. What are your problems in this area?<br>Can you think of any other relevant information that is missing relating to your body functions that has caused you problems in your daily life?
Body Structures	Thinking of your body, in which parts are your problem area?	Can you think of any other information that is missing relating to your body parts that has caused you problems in your daily life?
Activities & Participation	Thinking of your daily activities, in which areas do you	Learning and applying knowledge: This area involves all aspects of learning new

have problems with?	information and putting them into practice. What are your problems in this area?<br>General tasks and demands: This area involves all aspects of carrying out simple or complex tasks, organizing routines and handling stress. What are your problems in this area?<br>Communication: This area involves all aspects of communicating and carrying on conversations with others in different ways. What are your problems in this area?<br>Mobility: This area involves all aspects of moving, manipulating objects, walking or running and driving or using different kinds of transportation. What are your problems in this area?<br>Self-care: This area includes all aspects involving what you would usually do to take care of your body and your health, such as washing and drying oneself, dressing, eating and drinking. What are your problems in this area?<br>Domestic life: This area involves all aspects of finding a place to live, acquiring food and clothes, doing household chores and assisting others. What are your problems in this area?<br>Interpersonal interactions and relationships: This area involves all aspects of relating with others in different contexts, including family members, friends and strangers. What are your problems in this area?<br>Major life areas: This area involves all aspects of education, employment and economy. What are your problems in this area?<br>Community, social and civic life: This area involves all aspects of what you would do in your free time such as hobbies, religion, politics and community life. What are your problems in this area?<br>Can you think of any relevant information that is missing regarding daily activities

		and participation that has caused you problems in your daily life?
Environmental Factors	Thinking of your environment and your living conditions, what barriers or difficulties have you experienced?	Products and technology: This area involves all aspects of products and technology that you use in your daily life. What barriers or difficulties do you experience?<br>Support and relationships: This area involves all aspects of people and animals that provide you support at home, at work or at school (such as family members and professionals). What barriers or difficulties do you experience?<br>Attitudes: This area involves all aspects of other people's opinions and beliefs regarding yourself or your illness. What barriers or difficulties do you experience?<br>Services, systems and policies: This area involves all aspects of services (programs to meet the needs of individuals), systems (designed to organize, control and monitor services) and policies (rules of governments) of the society. What barriers or difficulties do you experience?<br>Can you think of any other environmental factors that are barriers or difficulties in your life?
Environmental Factors	Thinking of your environment and your living conditions, what do you find that is helpful or supportive?	Products and technology: This area involves all aspects of products and technology that you use in your daily life. What do you find that is helpful or supportive?<br>Support and relationships: This area involves all aspects of people and animals that provide you support at home, at work or at school (such as family members and professionals). What do you find that is helpful or supportive?<br>Attitudes: This area involves all aspects of other people's opinions and beliefs regarding yourself or your illness. What do you find that is helpful or supportive?<br>Services, systems and policies: This area involves all aspects of services (programs to meet the needs of individuals), systems (designed to organize, control and monitor

		services) and policies (rules of governments) of the society. What do you find that is helpful or supportive?<br>Can you think of any other environmental factors that have been helpful or supportive for you?
Personal Factors	Thinking of yourself and your personal characteristics, what are the important characteristics with handling your illness?	

Table S2.

Categories included in the Comprehensive ICF-CS for schizophrenia that were reported across focus groups.

ICF code and category title	Equatorial Guinea	India	Malaysia	Spain	Number of focus groups reporting the category
Focus groups conducted	$n = 1$	$n = 2$	$n = 3$	$n = 2$	
Body functions					
b114 Orientation functions	1	1	2	1	5
b117 Intellectual functions		2	2	2	6
b122 Global psychosocial functions (B)		2	3	2	7
b130 Energy and drive functions (B)	1	2	3	2	8
b134 Sleep functions	1	2	3	2	8
b140 Attention functions (B)		2	3	1	6
b144 Memory functions	1	2	3	2	8
b147 Psychomotor functions	1	2	2	1	6
b152 Emotional functions (B)	1	2	3	2	8
b156 Perceptual functions (B)	1	2	3	2	8
b160 Thought functions (B)	1	2	3	2	8
b164 Higher-level cognitive functions (B)	1	2	3	2	8
b180 Experience of self and time functions (B)	1	2	3	2	8
b330 Fluency and rhythm of speech functions	1	2	3	1	7

b530 Weight maintenance functions	1	2	3	2	8
b640 Sexual functions	1	1	2	1	5
b765 Involuntary movement functions	1	1	3	1	6
Activities and participation					
d155 Acquiring skills (B)	1	2	2	1	6
d160 Focusing attention		2	3	2	7
d163 Thinking	1	2	3	2	7
d166 Reading	1	2	2	1	6
d175 Solving problems (B)			1		1
d177 Making decisions		1	1		2
d210 Undertaking a single task	1	2	2	2	7
d220 Undertaking multiple tasks	1	2	3	1	7
d230 Carrying out daily routine (B)	1	2	3	2	8
d240 Handling stress and other psychological demands (B)	1	1	3	2	7
d310 Communicating with – receiving – spoken messages	1	2	3	1	7
d315 Communicating with – receiving – nonverbal messages	1	2	2		5
d330 Speaking	1	2	3	1	7
d335 Producing nonverbal messages	1	2	3		6
d350 Conversation	1	2	3	1	7
d470 Using transportation	1	2	3	2	8
d475 Driving		2	3	1	6
d510 Washing oneself	1	2	3	2	8
d520 Caring for body parts	1	1	3	2	7
d540 Dressing	1	2		2	5
d570 Looking after one’s health (B)	1	2	3	2	8
d610 Acquiring a place to live	1		2	1	4
d620 Acquisition of goods and services	1	2	3	2	8
d630 Preparing meals	1	1	2	1	5
d640 Doing housework	1	2	3	2	8
d650 Caring for household objects		2	3	1	6
d660 Assisting others	1		2	1	4
d710 Basic interpersonal interactions (B)	1	2	2	2	7
d720 Complex interpersonal interactions (B)	1	2	3	2	8

d730 Relating with strangers		2	3	2	7
d740 Formal relationships	1	2	2	2	7
d750 Informal social relationships	1	2	2	2	7
d760 Family relationships (B)	1	2	3	2	8
d770 Intimate relationships		2	1	2	5
d820 School education	1	2	3	2	8
d825 Vocational training	1	2	3	2	8
d830 Higher education	1	2	3	2	8
d840 Apprenticeship (work preparation)	1	1			2
d845 Acquiring, keeping and terminating a job (B)	1	2	2	2	7
d850 Remunerative employment	1	2	3	1	7
d855 Non-remunerative employment	1	2	2	1	6
d860 Basic economic transactions	1	2	3	2	8
d865 Complex economic transactions	1	2	3		6
d870 Economic self-sufficiency	1	2	3	2	8
d910 Community life (B)	1	1	3	1	6
d920 Recreation and leisure	1	2	3	2	8
d930 Religion and spirituality		1	3	2	6
d950 Political life and citizenship	1	2	3	1	7
Environmental factors					
e110 Products or substances for personal consumption	1	2	3	2	8
e125 Products and technology for communication	1	2	3	2	8
e130 Products and technology for education		2	3	2	7
e165 Assets	1	2	3	2	8
e310 Immediate family (B)	1	2	3	2	8
e315 Extended family	1	2	3	2	8
e320 Friends	1	2	3	2	8
e325 Acquaintances, peers, colleagues, neighbours and community members	1	2	3	2	8
e330 People in position of authority	1	2	3	1	7
e340 Personal care providers and personal assistants		2	3	2	7

e355 Health professionals (B)	1	2	3	2	8
e360 Other professionals	1	2	3	2	8
e410 Individual attitudes of immediate family members (B)	1	2	3	2	8
e415 Individual attitudes of extended family members	1	2	3	2	8
e420 Individual attitude of friends	1	2	3	2	8
e425 Individual attitudes of acquaintances, peers, colleagues, neighbours and community members	1	2	3	2	8
e430 Individual attitudes of people in position of authority	1	2	3	2	8
e440 Individual attitudes of personal care providers and personal assistants		2	3	1	6
e450 Individual attitudes of health professionals (B)	1	2	3	2	8
e455 Individual attitude of health-related professionals			1		1
e460 Societal attitudes (B)	1	2	3	2	8
e465 Social norms, practices and ideologies		2	3	2	7
e525 Housing services, systems and policies	1	2	3	2	8
e545 Civil protection services, systems and policies	1	2	3	2	8
e550 Legal services, systems and policies	1	2	3	2	8
e555 Associations and organizational services, systems and policies	1	2	3	2	8
e560 Media services, systems and policies	1	2	3	2	8
e570 Social security services, systems and policies (B)	1	2	3	2	8
e575 General social support services, systems and policies	1	2	3	2	8
e580 Health services, systems and policies (B)	1	2	3	2	8
e585 Education and training services, systems and policies	1	2	3	2	8
e590 Labour and employment services, systems and policies	1	2	3	2	8

Note. B: Categories included in the Brief ICF-CS for schizophrenia

Table S3.

Additional ICF categories reported across focus groups.

ICF code and category title	Equatorial Guinea	India	Malaysia	Spain	Number of focus groups reporting the category
Focus groups conducted	*n* = 1	*n* = 2	*n* = 3	*n* = 2	
Body functions					
b110 Consciousness functions	1	1	2		4
b126 Temperament and personality functions	1	2	3	1	7
b167 Mental functions of language	1	2	3	2	8
b172 Calculation functions		2	1		3
b176 Mental function of sequencing complex movements				1	1
b210 Seeing functions		2	1	1	4
b230 Hearing functions		2			2
b240 Sensations associated with hearing and vestibular function			1	1	2
b265 Touch function			1		1
b270 Sensory functions related to temperature and other stimuli		2	1		3
b280 Sensation of pain	1	2	1	1	6
b310 Voice functions		1	1		2
b320 Articulation functions				1	1
b340 Alternative vocalization functions			2		2
b410 Heart functions		1	1	1	3
b415 Blood vessel functions			1	1	2
b420 Blood pressure functions			2		2
b435 Immunological system functions				2	2
b440 Respiration functions		1	1	1	3
b455 Exercise tolerance functions	1	1	2	2	6
b460 Sensations associated			1		1

with cardiovascular and respiratory functions					
b510 Ingestion functions		1		2	3
b515 Digestive functions		2	2		4
b525 Defecation functions		2		1	3
b535 Sensations associated with the digestive system		2		2	4
b540 General metabolic functions		1	1	1	3
b545 Water, mineral and electrolyte balance functions		1	1		2
b550 Thermoregulatory functions		2	1	1	4
b555 Endocrine gland functions			1		1
b620 Urination functions		1	3	1	5
b650 Menstruation functions			1		1
b660 Procreation functions		1			1
b710 Mobility of joint functions			1		1
b715 Stability of joint functions			1		1
b730 Muscle power functions	1	1	2		4
b735 Muscle tone functions			1		1
b750 Motor reflex functions	1		1	2	4
b755 Involuntary movement reaction functions			1	1	2
b760 Control of voluntary movement functions	1	2	3	2	8
b770 Gait pattern functions		1	2	1	4
b780 Sensations related to muscles and movement functions	1		1	1	3
Body structure					
s110 Structure of brain	1	2			3
s140 Structure of sympathetic nervous system			1		1
s150 Structure of parasympathetic nervous system			1		1
s320 Structure of mouth		1	1		2
s410 Structure of cardiovascular system		2	1	1	4
s430 Structure of respiratory system			1		1

s520 Structure of esophagus		1			1
s530 Structure of stomach		2	2	2	6
s540 Structure of intestine		1			1
s630 Structure of reproductive system		1	1		2
s710 Structure of head and neck region		1			1
s730 Structure of upper extremity	1		2		3
s750 Structure of lower extremity		1	1		2
s760 Structure of trunk		1	1	1	3
s770 Additional musculoskeletal structures related to movement		1	3	1	5
s810 Structure of areas of skin		1			1
Activities and participation					
d110 Watching		2	1	1	4
d115 Listening		1	2		3
d170 Writing	1	2	2	2	7
d360 Using communication devices and techniques	1	2	2	1	6
d410 Changing basic body position		2	1	1	4
d415 Maintaining a body position		2	1	1	4
d430 Lifting and carrying objects			1	1	2
d440 Fine hand use			2		2
d450 Walking	1	2	1	1	5
d455 Moving around			2	1	3
d460 Moving around in different locations		1	1		2
d550 Eating	1	2	3	1	7
d560 Drinking		2		1	3
d810 Informal education		2	3	2	7
d940 Human rights	1	1	3	2	7
Environmental factors					
e115 Products and technology for personal use in daily living	1	2	3	2	8
e120 Products and technology for personal indoor and outdoor mobility and transportation	1	1	2	1	5

e135 Products and technology for employment	1	2	3	2	8
e140 Products and technology for culture, recreation and sport	1	2	3	2	8
e145 Products and technology for the practice of religion and spirituality	1	1	3		5
e150 Design, construction and building products and technology of buildings for public use		1			1
e155 Design, construction and building products and technology of buildings for private use		1			1
e160 Products and technology of land development	1		2	1	4
e210 Physical geography	1			1	2
e215 Population			1	2	3
e220 Flora and fauna			1		1
e225 Climate		1	1		2
e235 Human-caused events		1	2		3
e260 Air quality			1		1
e345 Strangers				1	1
e350 Domesticated animals		1	2	1	4
e515 Architecture and construction services, systems and policies	1	1	3	1	6
e520 Open space planning services, systems and policies	1	2	3	2	8
e530 Utilities services, systems and policies	1	2	3	2	8
e535 Communication services, systems and policies	1	2	3	2	8
e540 Transportation services, systems and policies	1	2	3	2	8
e565 Economic services, systems and policies	1	2	3	2	8
e590 Labour and employment services, systems and policies		1			1
e595 Political services, systems and policies	1	2	3		6
